# A new concept in NF2 pharmacotherapy: targeting fatty acid synthesis

**DOI:** 10.18632/oncoscience.417

**Published:** 2018-06-23

**Authors:** Dina S. Stepanova, Lanita Braun, Jonathan Chernoff

**Affiliations:** Fox Chase Cancer Center, Philadelphia, PA, 19111, USA

**Keywords:** neurofibromatosis 2, small molecule inhibitors, fatty acid synthesis

Neurofibromatosis type 2 (NF2) is an autosomal dominant disorder characterized by the development of multiple CNS tumors, most notably schwannomas and meningiomas. Mutational inactivation of the NF2 gene is found in most sporadic and inherited schwannomas, but the molecular mechanisms underlying neoplastic changes in schwannoma cells remain unclear, and effective medical treatments have not been identified. The protein encoded by the NF2 gene, Merlin, is not an enzyme and is either damaged or lost altogether in patients with the NF2 syndrome, and thus not directly targetable by small molecule inhibitors.

Merlin regulates multiple cellular functions that affect proliferation and survival. Currently, three targeted therapeutic approaches are being investigated for potential use in NF2: angiogenesis inhibitors, EGFR inhibitors, and MTOR inhibitors. While the use of such targeted agents has shown promise in early trials, the redundancy of signalling cascades in Merlin-null cells suggests a need to identify additional therapeutic concepts and targets in this disorder [1].

One potential new strategy in cancer is to exploit metabolic differences between neoplastic and normal cells. We recently performed a comparative study of metabolic properties of Nf2-deficient and wild-type (WT) cells to identify the differences potentially caused by the loss of Nf2 [2]. We profiled metabolism in WT and Nf2-deficient MEFs and in Nf2-WT (FC912) and Nf2-deficient (FH912) murine Schwann cells. We found several changes in the metabolic profile of Nf2-deficient cells, particularly, elevation of TCA cycle metabolites, long-chain and branched fatty acids, and glutamate metabolites, indicating increased energy demand in the Nf2-deficient cells. However, the most profound metabolic change was a marked increase in fatty acid levels.

Further investigation showed that fatty acid synthase (FASN) inhibitors selectively killed Nf2-deficient MEFs, schwannoma, and meningioma cells. We showed that such cells were sensitive to the FASN inhibitors cerulenin, C75, and GSK2194069, as well as anti-Fasn siRNAs. The effect was observed in MEF Nf2−/− cells, Nf2-deficient schwannoma cells (SC4-9), and Nf2-deficient schwannoma cells (RT4), as well as human cells obtained from Nf2-deficient schwannoma and meningioma samples. Merlin re-expression in SC4-9 cells rendered them insensitive to FASN inhibitors, confirming that such sensitivity depends on Merlin expression levels. Both cerulenin and GSK2194069 also inhibited tumour growth in mouse xenograft studies with SC4-9 cells.

**Figure 1:**
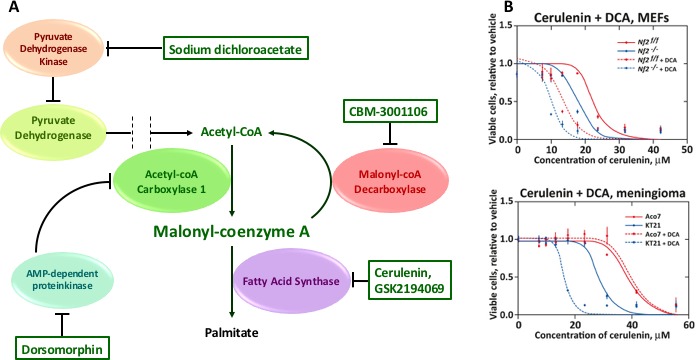
Differential effects of manipulating malonyl-coA levels in Nf2-null cells **(A)** Small molecules that increase malonyl-CoA levels by increasing its synthesis or by reducing its catabolism; **(B)** Effect of chemical inhibition of PDK on cerulenin toxicity. The PDK inhibitor sodium dichloroacetate (50 mM) was added together with the indicated concentrations of cerulenin, 4 hours after cell seeding, and cells were then incubated for 48 hours. Experiments were repeated 3 times. Mean values and 95% confidence intervals are shown. MEF Nf2−/− - Nf2-knockout murine embryonic fibroblasts, MEF Nf-2f/f – wild-type murine embryonic fibroblasts, KT21 – malignant NF2-deficient human meningioma cells, Aco7 – normal human arachnoid cells.

Cerulenin toxicity is thought to be mediated by an accumulation of fatty acid synthesis intermediate products due to the blockade of FASN. FASN catalyzes the production of palmitic acid, an essential building block for long-chain fatty acids. By inhibiting FASN, cerulenin blocks the malonyl-CoA condensation step of fatty acid synthesis, causing a deficit in palmitic acid as well as an accumulation of malonyl-CoA. The toxicity of malonyl-CoA in cancer cells is thought to be mediated by inhibition of carnitine palmitoyltransferase 1-regulated fatty acid β-oxidation, in turn promoting the accumulation of the sphingolipid ceramide followed by the induction of the pro-apoptotic genes such as BNIP3, TRAIL and DAPK2, effectors in the ceramide-mediated apoptotic pathway [3]. To address the potential toxicity of elevated malonyl-coA in Nf2−/− cells, we knocked down acetyl-CoA carboxylase 1 (ACC1), which catalyzes production of malonyl-CoA from acetyl-CoA to provide building blocks for FASN (Figure 1A). In Nf2−/− cells, knock-down of ACC1 greatly reduced the inhibitory effects of cerulenin- and GSK2194069, respectively. Conversely, knockdown of malonyl-CoA decarboxylase, which catalyzes the conversion of malonyl-CoA back to acetyl-CoA, increased the sensitivity of both cell lines to cerulenin and GSK2194069. Treatment with an ACC activator, 5-iodotubercidin, which blocks an AMP kinase mediated inhibitory phosphorylation of ACC1 [4], caused an increase in cerulenin toxicity in Nf2f/f cells, but had little effect on cerulenin toxicity in Nf2−/− cells. Similarly, increasing malonyl-CoA by activating pyruvate dehydrogenase with dichloroacetate was also selectively toxic to Nf2−/− MEFs and meningioma cells (Figure 1B).

The FASN inhibitor cerulenin has been shown to inhibit the proliferation of colon, breast, and prostate cancer cells [5]. The sensitivity of certain cancer cells to this drug may be due to higher fatty acid synthesis levels compared to normal cells [6]. Our findings showed that, despite their benign nature, Merlin-null cells have markedly elevated levels of fatty acid synthesis, and displayed significantly higher levels of FASN and also ACC1 and 2, the enzymes that catalyze production of malonyl-CoA from acetyl-CoA, as well as low levels of ACC phosphorylation, indicating high activity of this enzyme [2, 7]. The higher level of ACC expression is consistent with the pronounced rescue effect of TOFA, an ACC inhibitor, on cerulenin-treated Nf2−/− MEFs [2]. Interestingly, FASN inhibitors have previously been shown to reduce proliferation of human malignant mesothelioma cells, which are often characterized by loss of function mutations in NF2 [8]. In addition, Haase et al. reported that FASN expression was elevated in 70% of atypical grade II and anaplastic grade III meningiomas, and that treatment with cerulenin significantly decreased NF2-null meningioma cell survival in vitro and reduced tumour volumes in xenografts [9]. These results, combined with our data in NF2-null MEFs and Schwann cells, suggest that changes in lipid synthesis may be a general function in cells lacking Merlin and suggest that FASN inhibitors pose promising agents for neurofibromatosis II pharmacotherapy.
